# News Items

**Published:** 1885-05

**Authors:** 


### News Items.

The Illinois State Medical Society, at its annual session, recommended to the Legislature of this State the enactment of the following bill, prepared by the committee appointed by the society for that purpose:

*A Bill for an Act to provide for the Commitment of the Insane.—Be it enacted.* First, That the judges of the courts of each judicial district shall, jointly, nominate to the Governor, within sixty days next after the passage of this Act, two physicians in each county best qualified to act as Commissioners of Insanity.

Second, That the Governor, within thirty days next after the reception of such nomination, shall appoint the said two physicians to be Commissioners of Insanity in and for each of said counties respectively.

Third, That the said two Commissioners so appointed, together with the Judge of the County Court, in and for each of said counties, respectively, constitute the Board of Commissioners of Insanity for said county.

Fourth, That the said Board of Commissioners have exclusive and complete jurisdiction of all matters pertaining to the commitment and custody of the insane, and power and authority adequate to the determination thereof.

Fifth, That affidavit of alleged insanity be made to the Clerk of the County—*ex-officio* Clerk to the Board of Commissioners of Insanity—to be reported by him to the Judge of the County Court—*ex-officio* President of the said Board—within twenty-four hours of the date of the said affidavit.

Sixth, That the alleged insane person be examined by each and all of the said Commissioners, privately, publicly, separately or jointly, as may be deemed, by each, necessary to the determination of the truth.

Seventh, That insanity be determined only by unanimous decision of the said Board.

Eighth, That, in case of disagreement, the minority may demand, and the Board must grant, a re-examination.

Ninth, That the said Board, having determined the insanity of any one, shall order his (or her) commitment, if curable, to a State hospital for the insane, if incurable, to a county asylum, or to such other custody as may seem best adapted to the circumstances of any special case.

Tenth, That each of said Commissioners, so appointed, receive the sum of —— dollars for each and every case of alleged insanity examined by him, and in addition thereto the sum of —— —— for each and every mile necessarily traveled by him to and from the places of holding said examination, the same to be paid as fees to jurors hitherto.

Eleventh, That the term of service of one of said Commissioners, so appointed, be three years, to be determined by lot; that of all others to be five years—in order that a majority of said Board may always consist of old members.

*Provided*, That nothing in this Act shall be construed to prevent the trial by jury, as at present, of any alleged insane person who may, either personally or by his or her friends, elect that mode of examination; and that for all such cases the statutes now in force shall continue effective.

The law now in force in this State, in its practical working, does violence to the feelings of many persons, charged with being insane, by being brought into open court and tried by jury. No doubt the shock thus sustained is prejudicial to their welfare, and some more humane method of proceeding should be adopted. The bill proposed, if enacted by the present Legislature, as it is hoped it will be, will do much to relieve such unfortunates from the evils attendant upon jury trial, and yet leaves the right of trial by jury in cases where for any reasons it may be demanded.

